# “Suture-first” endoscopic transection of a postoperative rectal mucosal bridge: a safety strategy to prevent adverse events

**DOI:** 10.1055/a-2839-7267

**Published:** 2026-04-15

**Authors:** Abdeldjalil Sais, Salah Merar, Cedric Scheiwe, Jérôme Rivory, Jérémie Jacques, Jean Grimaldi, Mathieu Pioche

**Affiliations:** 1 639305 Department of Gastroenterology, Groupe Hospitalier Portes de Provence (GHPP), Montélimar, France; 2 56485 Department of Gastroenterology and Endoscopy, Centre Hospitalier de Roanne, Roanne, France; 3 36609 Department of Visceral, Digestive and Bariatric Surgery, Hôpital Edouard Herriot, Hospices Civils de Lyon, Lyon, France; 4 Department of Gastroenterology and Endoscopy, Hôpital Edouard Herriot, Hospices Civils de Lyon, Lyon, France; 5 Gastroenterology and Endoscopy Unit, Dupuytren University Hospital, Limoges, France

Postoperative colorectal luminal abnormalities are typically interpreted as strictures but intraluminal mucosal bridges (synechiae) can create a “double-lumen” pseudo-stenosis that may impair functional outcomes before stoma reversal 1 . We describe a preventive endoscopic strategy to reduce the risk of adverse events during the transection of such a large bridge at the middle of the colorectal anastomosis.

**Fig. 1:**
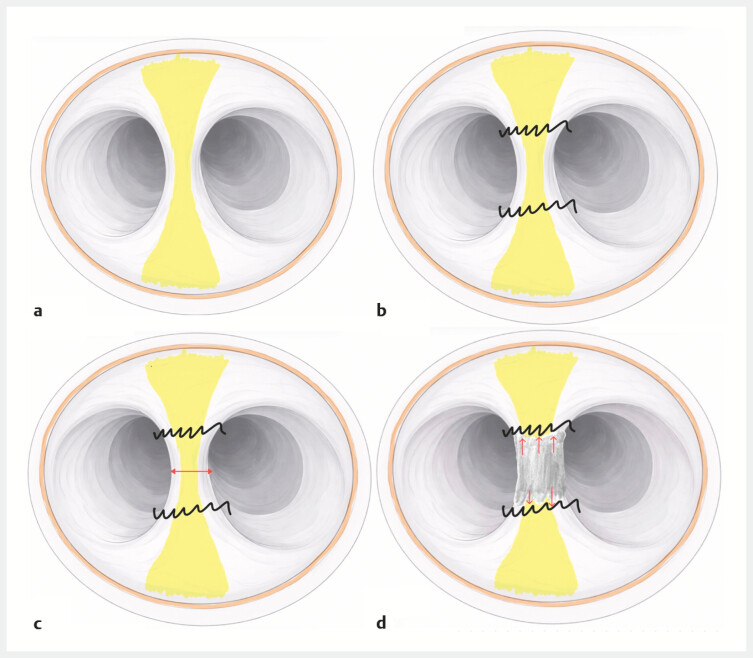
A suture-first strategy for the endoscopic division of rectal synechia. **a** An initial endoscopic view of rectal synechia. **b** Pre-emptive suturing at the bridge base to secure underlying structures. **c** Controlled midline transection. **d** Clip closure of the resection margins with restoration of a single lumen.

A 68-year-old woman was evaluated prior to the reversal of a diverting ileostomy after sigmoid surgery for diverticular peritonitis. Although a postoperative anastomotic fistula had healed, pre-reversal rectoscopy revealed a mid-rectal mucosal bridge dividing the lumen into two channels ( Fig. 1 **a** ). Because the bridge pillars were located in a postoperative field, simple transection was considered to carry a risk of pillar-wall injury with bleeding or perforation, potentially leading to a leakage in the peri digestive space 2 .

The procedure was performed under general anesthesia. A “suture-first” strategy was applied using the SutuArt through-the-scope needle-holder system (Olympus, Tokyo, Japan [ Video 1 ]). First, sutures were placed to close both bridge pillars and secure hemostasis ( Fig. 1 **b** ). Suturing appeared to be a simple way to turn around the pillar and catch the full area tightened. The bridge was then sectioned at its midpoint between the two sutured pillars using an electrosurgical knife ( Fig. 1 **c** ). Finally, five clips were applied to reinforce both sides of the transection site on the suture wire to avoid further slipping ( Fig. 1 **d** ).

A postoperative rectal mucosal bridge: pillar closure with SutuArt sutures, midpoint transection, and reinforcement with five clips, restoring a single wide lumen without bleeding or perforation.Video 1

The maneuver immediately restored a single, wide rectal lumen. No bleeding or perforation occurred. The patient received prophylactic amoxicillin–clavulanic acid for 5 days and was discharged the following day with a normal diet. Three weeks later, healing was confirmed with a rectoscope before stoma reversal.

This video illustrates a reproducible “close-cut-reinforce” sequence that improves safety when treating postoperative rectal mucosal bridges endoscopically 3 .

Endoscopy_UCTN_Code_CCL_1A
